# Associations between gentrification, census tract-level socioeconomic status, and cycling infrastructure expansions in Montreal, Canada

**DOI:** 10.1016/j.ssmph.2024.101637

**Published:** 2024-02-18

**Authors:** Behzad Kiani, Benoit Thierry, Philippe Apparicio, Caislin Firth, Daniel Fuller, Meghan Winters, Yan Kestens

**Affiliations:** a Centre for Clinical Research, Faculty of Medicine, The University of Queensland, Brisbane, Australia; b Centre de Recherche en Santé Publique, Université de Montréal, 7101, Avenue du Parc, Montréal, H3N 1X9, Canada; c Department of Applied Geomatics, Université de Sherbrooke, 2500, boulevard de l'Université, Sherbrooke, J1K 2R1, Canada; d Department of Psychiatry and Behavioral Sciences, University of Washington School of Medicine, 1959 NE Pacific St, Seattle, WA 98195, USA; e University of Saskatchewan, 105 Administration Place, Saskatoon, S7N 5A2, Canada; f Faculty of Health Sciences, Simon Fraser University, 8888 University Drive, Burnaby, V5A 1S6, Canada

**Keywords:** Cycling infrastructure, Equity, Visible minority, Gentrification, Material deprivation, Hurdle modeling

## Abstract

**Background:**

Cycling infrastructure investments support active transportation, improve population health, and reduce health inequities. This study examines the relationship between changes in cycling infrastructure (2011–2016) and census tract (CT)-level measures of material deprivation, visible minorities, and gentrification in Montreal.

**Methods:**

Our outcomes are the length of protected bike lanes, cyclist-only paths, multi-use paths, and on-street bike lanes in 2011, and change in total length of bike lanes between 2011 and 2016 at the CT level. Census data provided measures of the level of material deprivation and of the percentage of visible minorities in 2011, and if a CT gentrified between 2011 and 2016. Using a hurdle modeling approach, we explore associations among these CT-level socioeconomic measures, gentrification status, baseline cycling infrastructure (2011), and its changes (2011–2016). We further tested if these associations varied depending on the baseline level of existing infrastructure, to assess if areas with originally less resources benefited less or more.

**Results:**

In 2011, CTs with higher level of material deprivation or greater percentages of visible minorities had less cycling infrastructure. Overall, between 2011 and 2016, cycling infrastructure increased from 7.0% to 10.9% of the road network, but the implementation of new cycling infrastructure in CTs with no pre-existing cycling infrastructure in 2011 was less likely to occur in CTs with a higher percentage of visible minorities. High-income CTs that were ineligible for gentrification between 2011 and 2016 benefited less from new cycling infrastructure implementations compared to low-income CTs that were not gentrified during the same period.

**Conclusion:**

Montreal's municipal cycling infrastructure programs did not exacerbate socioeconomic disparities in cycling infrastructure from 2011 to 2016 in CTs with pre-existing infrastructure. However, it is crucial to prioritize the implementation of cycling infrastructure in CTs with high populations of visible minorities, particularly in CTs where no cycling infrastructure currently exists.

## Highlights

• Census tracts (CTs) with higher material deprivation or visible minorities had less cycling infrastructure in 2011 and 2016.
• In CTs without cycling infrastructure, new installations were less likely in CTs with more visible minorities.
• In CTs with cycling infrastructure, material deprivation and visible minorities were not related to infrastructure increase.
• In CTs with cycling infrastructure, high-income CTs benefited less from infrastructure expansions than non-gentrified CTs.
• Hurdle modeling can reveal equity disparities in cycling infrastructure by CTs with/without infrastructure.

## 1 Introduction

Investments in cycling infrastructure by cities can support healthy lifestyles and improve accessibility to active transportation infrastructure (Smith et al., 2017). However, these investments should be prioritized in neighborhoods that lack infrastructure, that is, often in areas of lower socioeconomic status (SES) or areas with more visible minorities, or else communities who need it most may not benefit as expected from these improvements. People with less access to bike paths and other cycling facilities might have less chance to engage in active transportation, contributing to less active lifestyles and worse health. This is called "exacerbating environmental injustice", which means making an unfair situation worse (Braun, 2021; Mölenberg et al., 2019; Smith et al., 2017). Therefore, it is essential to comprehensively document and understand where increases in cycling infrastructure are occurring and if and how they impact inequities in accessibility to active transportation infrastructure.

The relationship between access to cycling infrastructure and the socioeconomic landscape of urban areas is multifaceted, engaging with a range of theoretical underpinnings, discussing various demographic, geographic, and economic factors that could affect active travel demands (Pratt et al., 2012; Pucher & Buehler, 2008; Evaluating Active Transport Benefits and). For instance, densely populated neighborhoods with limited access to public transportation, may witness an increase in cycling as a primary mode of transport. Additionally, economically disadvantaged communities may heavily rely on cycling as an affordable means of commuting (Stein, 2011). On the other hand, the arrival of new, high SES residents, as part of the process of gentrification in some urban neighborhoods may have led to a shift in preferences towards sustainable transportation options (Bruno, 2022). To further dissect this intricate relationship, we engage in socio-spatial analyses. These analyses offer insight into how the social and economic dynamics within urban areas intersect with the availability and accessibility of cycling infrastructure.

Area-level SES indicators can be used to conduct socio-spatial analyses showing how social and economic conditions of urban areas may be linked to differential levels of access to active transportation. Specifically, the level of material deprivation, and the percentage of visible minorities, may be associated with the availability of cycling infrastructure, at the local area Census tract (CT) level (Stehlin, 2015; Vidal Tortosa et al., 2022). Material deprivation has been used in many Canadian public health studies to document social health inequities (de Oliveira et al., 2021; Loignon et al., 2015; Smithman et al., 2018). It is a factor score summarizing Census measures of income, employment, housing, and education (Pampalon et al., 2012). Visible minorities have also been reported to have less access to cycling infrastructure (Hosford et al., 2022; Lowan-Trudeau et al., 2020; Vaswani et al., 2023). The notion of visible minority, as used in this study, refers to self-reported data about ethnicity collected by Statistics Canada and denotes individuals who report identifying as being not white and not Indigenous (Government of Canada SC, 2021).

Implementation of cycling infrastructure has also been linked to gentrification (Padeiro et al., 2019), a process in which previously deteriorating, under-resourced neighborhoods undergo further investment and in-migration of increasingly affluent new residents (Tulier et al., 2019). Gentrification is often a complex and multifaceted process that cannot be attributed solely to cycling infrastructure or any single factor (Padeiro et al., 2019). Cycling infrastructure enhancements may contribute to gentrification by increasing property values and attracting higher-income residents (Hoffmann, 2013), but they can also benefit low-income communities by enhancing transportation access and reducing traffic-related health externalities (Noyes et al., 2014). The connection between cycling infrastructure and gentrification is still being studied and debated since the relationship may depend on factors such as location, timing, and local policy priorities (Padeiro et al., 2019).

Research conducted in various countries has yielded mixed findings. For example, studies in South American settings have shown that low-income communities in places like Bogotá, Rio de Janeiro, and Curitiba face a notably lower level of accessibility to bike lanes (Equality in Access, 2015; Parra et al., 2018; Tucker & Manaugh, 2018). In contrast, a study in England showed that deprived areas have higher cycling infrastructure and cyclability levels compared to non-deprived areas (Tortosa et al., 2021). A significant difference between the most and least deprived areas in terms of the presence of bike lanes has been also observed in Portugal (Padeiro, 2022). In Braun et al.'s study examined data between 2012 and 2016, it was observed that block groups characterized by specific forms of disadvantage, such as lower educational attainment, a higher proportion of Hispanic residents, and lower composite SES, exhibited reduced accessibility to bike lanes. However, this pattern was not observed in block groups facing different types of disadvantage, such as a higher proportion of black residents, lower income levels, and higher poverty rates (Braun et al., 2019). The absence of sociospatial equity considerations in most transportation plans of Canadian cities may limit the effectiveness of new cycling infrastructure implementation to reduce inequities (Doran et al., 2021). Current research in various Canadian cities has shown mixed findings regarding the distribution of cycling infrastructure, with some showing lower accessibility to cycling infrastructure in CTs with more children or people of certain ethnic backgrounds (Firth et al., 2001), and another showing more cycling infrastructure in lower-income CTs in Halifax, Victoria, and Kelowna (Winters et al., 2018).

Among Canadian cities, Montreal has a relatively extensive cycling infrastructure, a bike-sharing program (BIXI), and winter cycling lanes that also promote cycling during the cold months (Jarry & Apparicio, 2021). In 2020, a 42% increase from 2015 was observed in the number of adults who utilized bicycles for utility purposes occasionally or more often, representing two out of three adults or 600,000 people. Moreover, the increase in frequency of use is notable, with 350,000 Montreal residents cycling once a week or more in 2020, compared to 275,000 individuals five years earlier. This data firmly underscores cycling's integral role within Montreal's urban fabric (Pucher & Buehler, 2005; Québec). However, studies on cycling conducted in Montreal have focused on impacts on health outcomes and safety, but few studies have analyzed the evolution of the cycling network from a perspective of SES equity over time (Apparicio et al., 2016; Fuller et al., 2013; Hatzopoulou et al., 2013; Strauss et al., 2013). Houde et al. used a longitudinal design to examine the distribution of cycling infrastructure in different CTs over the quarter-century period from 1991 to 2016 (Houde et al., 2018). The study found that while access to cycling infrastructure was initially greater in CTs with more low-income populations, it became more evenly distributed over time. However, it is worth noting that the study employed separate modeling approaches for different years, combining elements of both longitudinal and repeated-measure cross-sectional designs, to measure the associations between cycling infrastructure and SES factors at specific points in time (Houde et al., 2018).

In relation to gentrification, research in the US has delved into the association between gentrification and the development of cycling infrastructure. Research demonstrated that gentrification was strongly associated with the subsequent installation of bicycling facilities rather than the reverse, suggesting that the installation of bicycling facilities did not occur prior to gentrification (Firth et al., 2001). Moreover, marginalized communities were less likely to attract substantial investment in cycling infrastructure unless privileged populations were present. This phenomenon persisted even when accounting for factors such as population density and distance to downtown, which are commonly considered as driving factors for urban cycling (Winters et al., 2018). In a recent study conducted in Montreal, an investigation into the association between gentrification from 2011 to 2016 and the expansion of cycling infrastructure in the same period revealed no significant associations. It is worth noting that the study specifically concentrated on gentrified and non-gentrified CTs, without taking into account high-income CTs, which are ineligible for gentrification (Kiani et al., 2023).

The mixed finding on the literature of cycling infrastructure and area level SES measures suggests that the equitable distribution of cycling infrastructure may be influenced by historical human settlement patterns and land use, making it city specific phenomenon. Cities are currently investing in cycling infrastructure, however it is unclear whether area-level SES inequalities in accessibility to cycling infrastructure are increasing or decreasing over time (Pucher & Buehler, 2008). Therefore, this study aims to investigate the association between CT-level SES measures, gentrification and cycling infrastructure expansions in the Montreal region over 2011–2016. We further test if these associations vary depending on the presence or absence of pre-existing cycling infrastructure.

## 2 Methods

### 2.1 Study area

**Fig. 1:**
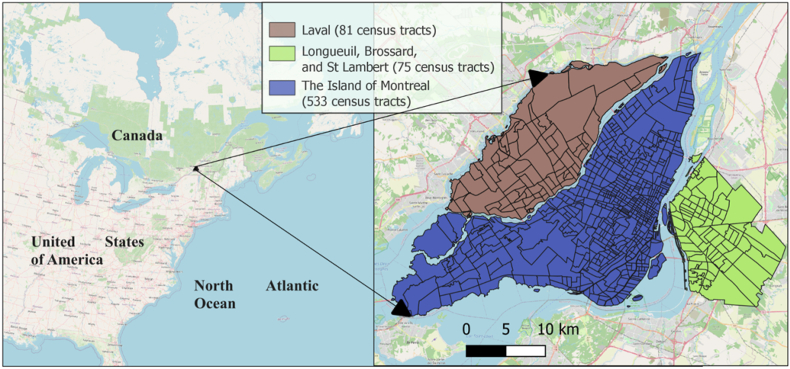
The geographical location of the study area on a global scale, along with the distribution of census tracts.

This is a retrospective study associated with **blinded for peer-review**. The study area includes Montreal Island (N = 533 Census tracts (CTs) in 2016) as well as a portion of the South (Longueuil, Brossard, and St Lambert, N = 65) and North shores (Laval, N = 91), for a total of 689 CTs in 2016. Fig. 1 displays the study area's geographical location on a global scale, along with the CTs distribution.

### 2.2 Data and variables

We used census data to measure two CT-level SES measures (the level of material deprivation, percentage of visible minorities) as well as gentrification status as predictor variables of cycling infrastructure. The selection of material deprivation, visible minorities, and gentrification as key variables in our analysis is underpinned by their relevance to the study's overarching objectives. Material deprivation, a critical socioeconomic factor, is often associated with disparities in access to resources, amenities, and opportunities. The Pampalon material and social deprivation scores are commonly used to study health inequities in Canada (Pampalon et al., 2012). In this study, we investigate how disparities in access to cycling infrastructure may intersect with broader socioeconomic inequities. The inclusion of minority populations is motivated by the need to explore equity in cycling infrastructure distribution, considering that minority communities may face unique challenges in accessing such infrastructure. By examining the presence and distribution of cycling infrastructure relative to minority populations, we assess whether disparities exist and whether interventions are needed to promote equitable access. Additionally, the analysis of gentrification allows us to uncover how changing neighborhood dynamics may influence the distribution of cycling infrastructure. Understanding the relationship between gentrification and cycling infrastructure provides insights into the evolving landscape of urban transportation options and potential consequences for diverse socioeconomic groups. Together, these variables contribute to a holistic understanding of the complex interplay between urban planning, social equity, and active transportation.

*Material deprivation in 2011:* We used the material deprivation score, which includes census variables of income, employment, and educational attainment. Raw data is provided at the dissemination area (ِDA) level, which we aggregated at the CT level, using a DA-population weighting, and classified into quintiles, from Q1 (least) to Q5 (most) materially deprived (Pampalon et al., 2012).

*Visible minorities in 2011:* A second indicator was the percentage of the population that reported being a visible minority (Government of Canada SC, 2021), which we again classified as quintiles.

*Gentrification.* The 2011–2016 gentrification status (gentrified, not gentrified, and non-eligible for gentrification) was assessed using Ding's measure, as provided in the GENUINE dataset (Firth et al., 2021). To be classified as gentrified, a CT needs to both be considered gentrifiable – that is, be below the median household income in the city – and witness an increase in both gross rent or home values and in the proportion of university-educated residents at the second time point that is above the median (Ding et al., 2016). If a CT's income is higher than the median household income at the baseline, it is considered non-eligible for gentrification and can be designated as a high-income CT.

*Cycling infrastructure:* Our data included protected bike lanes, cyclist-only paths, multi-use paths, and on-street bike lanes, as compiled by previous authors (Houde et al., 2018). Cycling infrastructure as the outcome variable was defined and modeled in two ways. First, as a binary variable of presence/absence of cycling infrastructure in any given CTs both in 2011 and 2016. Second, as a continuous variable of the percentage of road length with cycling infrastructure, in 2011 and 2016. This continuous variable was only calculated for CTs with pre-existing cycling infrastructure in 2011.

### 2.3 Analyses

#### 2.3.1 Descriptive analysis

We computed the median and the 25th and 75th percentiles of measures of cycling infrastructure at baseline (2011) and follow-up (2016), per quintile of material deprivation and percentage of visible minorities, and per gentrification status (non-gentrified, gentrified, and non-eligible for gentrification) for CTs with existing cycling infrastructure. We also computed the number of CTs with and without cycling infrastructure. Finally, we mapped the spatial distribution of cycling infrastructure and its 2011–2016 changes at the CT level to visualize the outcome variable.

#### 2.3.2 Modeling

We conducted Ordinary Least Squares (OLS) regression and have included the results in Supplementary File 1. However, due to the skewed distribution arising from the prevalence of zero values (CTs without any cycling infrastructure), the use of regular regression models, such as OLS, was deemed inappropriate for our analysis. Furthermore, OLS is not applicable to Spatial Mixed Effect Models, which we utilized to address the spatial component (modeled as a random effect). Therefore, we used a spatial hurdle modeling approach (Feng, 2021), which allows to model counts (percentage of road network with cycling infrastructure in our case) with a presence of numerous null values, similar to zero-inflated models. One component of the model evaluates how SES measures relate to the presence or absence of cycling infrastructure (zero model), whereas a second component evaluates how SES measures relate to the ratio of cycling infrastructure to street length among those CTs that have some cycling infrastructure (count model) (Neelon et al., 2013). The quintiles of the level of material deprivation and percentage of visible minorities as continues variables along with the gentrification status as categorical variable were used as predictors in both components of the model. Categorizing continuous data results in a loss of information. However, our approach, using quintiles, enhances the interpretation of the results and allows the identification of specific CTs were cycling infrastructure could be placed to reduce inequities.

A first series of models looked at cycling infrastructure in 2011 (baseline year), as per Equation (1):

Eq 1 $$Log\left(E\left({CI}_{2011}\right)\right)=\beta_{0}+\beta_{1}\times {SES}_{measure\;\left(2011\right)}+offset(\log\left(CT\_street\_network\_length\right)+u_{0}$$

Where CI_2011_ stands for cycling infrastructure in 2011, $\beta_{0}$ is the intercept, $\beta_{1}$ is the coefficient for the SES measure in 2011 and u_0_ is the spatial random effect. The offset represents the length of the street network in each census tract, excluding highways and maritime routes.

A second series of models assessed change in cycling infrastructure between 2011 and 2016. To do so, we modeled cycling infrastructure in 2016 while controlling for cycling infrastructure in 2011, and used the same main SES measures as predictors. We further tested an SES* 2011 cycling infrastructure interaction term to evaluate if cycling infrastructure at baseline modified the relation between CTs’ SES — and change in infrastructure between 2011 and 2016, as per Equation (2):

Eq 2 $$Log\left(E\left({CI}_{2016}\right)\right)=\beta_{0}+\beta_{1}\times {SES}_{measure\;\left(2011\right)}+\beta_{2}\times {CI}_{2011}+\beta_{3}\times {SES}_{measure\left(2011\right)}\times {CI}_{2011}+offset(\log\left(CT\_street\_network\_length\right)+u_{0}$$

Where *CI*_2016_ stands for the cycling infrastructure in 2016 (and to be interpreted as changes in cycling infrastructure between 2011 and 2016 as the model controls for *CI*_2011_), $\beta_{0}$ is the intercept, $\beta_{1}$ is the coefficient for the SES measure in 2011, $\beta_{2}$ is the coefficient for the cycling infrastructure in 2011 as a control variable, $\beta_{3}$ is the coefficient for the interaction between the SES measure and the cycling infrastructure in 2011, and *u*_0_ is the spatial random effect. The offset represents the length of the street network in each census tract, excluding highways and maritime routes.

We ran separate regression models for each SES factor (ie., the quintile level of material deprivation and percentage of visible minority minorities) and CTs’ gentrification status to mitigate issues of potential multicollinearity and facilitate interpretation of the results, as we could clearly observe the magnitude and direction of the relationship between each SES factor and cycling infrastructure without potential confusion from analyzing coefficients in a model with multiple independent variables.

Conditional spatial autocorrelation was defined by a contiguity matrix based on a Queen contiguity neighborhood rule, which means all CTs touching the border of the current CT are considered neighbors. Poisson models were chosen given the outcomes were all expressed as rates (count model), with the denominator being the total street length in hectometers, including both streets with and without cycling infrastructure (Coxe et al., 2009). Binomial distribution was used as the distribution probability model for the zero model in hurdle modeling approach.

We used R ver. 4.1.3 along with packages spaMM ver. 4.1.20 (Rousset & Ferdy, 2014) for spatial models and spdep ver. 1.2–5 (Bivand, 2022) for spatial neighborhood computation. Census data was retrieved using cancensus ver. 0.5.0 (Bergmann et al., 2021), spatial data extraction was handled using sf ver. 1.0–9 (Pebesma, 2018).

## 3 Results

### 3.1 Descriptive results

Table 1 shows that cycling infrastructure was present in 71% of CTs (487 of 689) in 2011, and in 84% of CTs (576 of 689) in 2016. CTs with lower levels of material deprivation or fewer visible minorities had more cycling infrastructure in 2011 and 2016. However, for both SES indicators, the Q1/Q5 ratio (ratio of cycling infrastructure in the least deprived CTs compared to the most deprived CTs) decreased between these two years, indicating a decrease in inequity in cycling infrastructure between 2011 and 2016.

**Table 1:** Descriptive statistics on cycling infrastructure across 689 census tracts in the study area, stratified by different social measures.

	Number of CTs with/without cycling infrastructure (% of CTs with cycling infrastructure)			Median % of street length with cycling infrastructure (Interquartile Range)		
	2011	2016	2016/2011	2011	2016	2016/2011
All CTs	487/202 (71)	576/113 (84)	1.18	7.0 (0–14.0)	10.9 (3.8–18.2)	1.56
Q1 Material Deprivation (2011)	99/36 (73)	110/25 (81)	1.11	7.6 (0–16.5)	11.5 (3.2–21)	1.51
Q5 Material Deprivation (2011)	75/60 (56)	96/39 (71)	1.27	1.3 (0–9.8)	8 (0–15.3)	6.15
Q1/Q5 material deprivation (2011)	1.3	1.14	0.88	5.85	1.44	0.12
Q1 Visible Minorities (2011)	98/37 (73)	116/19 (86)	1.18	8.1 (0–14.5)	12.9 (6–20.5)	1.59
Q5 Visible Minorities (2011)	74/61 (55)	92/43 (68)	1.24	1.1 (0–8.3)	6.6 (0–14.3)	6
Q1/Q5 visible minorities (2011)	1.33	1.26	0.95	7.36	1.95	0.26
Gentrified (2011–2016)	129/78 (62)	170/37 (82)	1.32	5.6 (0–14.5)	13.4 (3.9–20)	2.93
Not Gentrified (2011–2016)	142/80 (64)	176/46 (79)	1.23	5.8 (0–12.1)	9.6 (3–17.2)	1.66
Gentrified (2011–2016)/not gentrified (2011–2016)	0.97	1.04	1.07	0.97	1.40	1.44
Non-eligible for gentrification between 2011 and 2016	205/41 (83)	219/27 (89)	1.07	7.5 (1.7–13.6)	9.7 (3.8–16.1)	1.29
Non-eligible for gentrification between 2011 and 2016/non-gentrified between 2011 and 2016	1.29	1.13	0.88	1.29	1.01	0.78

“Gentrified” refers to CTs satisfying the definition between 2011 and 2016.

The non-eligible for gentrification/non-gentrified ratio decreased for both binary and continuous measures of cycling infrastructure, indicating that non-eligible for gentrification CTs obtained less cycling infrastructure compared to non-gentrified CTs. However, the gentrified/non-gentrified ratio increased, showing that gentrified CTs obtained greater increase in cycling infrastructure compared to non-gentrified CTs.

### 3.2 Spatial distribution and changes over time in cycling infrastructure

**Fig. 2:**
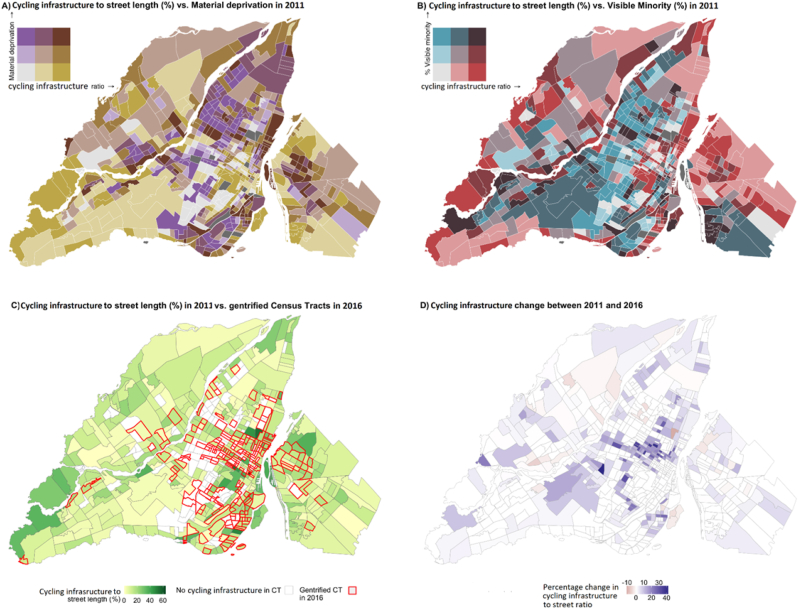
Spatial distribution of cycling infrastructure and socioeconomic factors A): cycling infrastructure vs. material deprivation in 2011 B): cycling infrastructure vs. visible minority in 2011 C): cycling infrastructure in 2011 vs. gentrified in 2016 D): cycling infrastructure change between 2011 and 2016.

Fig. 2 shows bivariate associations between cycling infrastructure and social factors in the study area. CTs without any cycling infrastructure are mostly located on Montreal Island in areas that are often more industrial or include a major airport (Fig. 2-C). In some CTs, cycling infrastructure decreased during the study period. However, most central CTs benefited from greater cycling infrastructure additions (Fig. 2-D).

### 3.3 Cycling infrastructure in 2011

Our models estimating how SES measures and gentrification related to 2011 cycling infrastructure (Table 2) show each additional quintile of material deprivation was associated with a 20% reduction in the likelihood of there being any cycling infrastructure in the CT (RR = 0.80, zero model). Each additional quintile of the percentage of visible minorities was linked to a 19% lower probability of there being any cycling infrastructure in the CT (RR = 0.81, zero model), and 5.6% less street length with cycling infrastructure (RR = 0.94, count model). Finally, there was no significant difference in cycling infrastructure presence nor quantity between gentrified and non-gentrified CTs and between non-eligible for gentrification and non-gentrified CTs.

**Table 2:** Association between SES measures and cycling infrastructure in 2011.

Models with a social measure as the outcome		Zero model (among all CTs)	Count model (among CTs with non-zero cycling infrastructure)
		N = 689	N = 576
Model 1- The quintile of material deprivation in 2011 as continues variable		0.80* CI = [0.65–0.97]	0.94 CI = [0.89–1.00]
Model 2- The quintile of percentage of visible minorities in 2011 as continues variable		0.81* CI = [0.67–0.97]	0.94* CI = [0.89–0.99]
Model 3- Gentrification status	Gentrified betw-een 2011 and 2016	1.05 CI = [ 0.59: 1.86]	1.11 CI = [0.93–1.33]
	Ineligible for gentrification between 2011 and 2016 (High-income CTs)	1.28 CI = [0.70–2.41]	0.99 CI = [0.84–1.17]

Statistically significant values based on 95% confidence intervals are denoted by an asterisk (*). CT refers to Census Tract. Zero and count models are based on hurdle modeling approach standing for the binary outcome (CTs with and without cycling infrastructure at baseline) and the continuous outcome (cycling infrastructure percentage among street length).

### 3.4 Cycling infrastructure change between 2011 and 2016

The change in the amount of cycling infrastructure between 2011 and 2016 was not associated with the 2011 levels of material deprivation nor the percentage of visible minorities (Table 3). However, the negative significant coefficient of the association between cycling infrastructure implementations and the percentage of visible minorities (zero model) shows CTs without infrastructure in 2011 had a lower probability of witnessing any implementation in 2016 if they had a higher percentage of visible minorities. Finally, the count model shows that non-eligible CTs for gentrification benefitted less from increases in cycling infrastructure than non-gentrified CTs. However, the significant interaction term showed that non-eligible CTs for gentrification with more cycling infrastructure at baseline benefited from greater increases in cycling infrastructure compared to non-eligible CTs for gentrification with less cycling infrastructure at baseline.

**Table 3:** Association between SES measures in 2011 and cycling infrastructure in 2016, controlling for cycling infrastructure in 2011 to reflect the change in cycling infrastructure between 2011 and 2016.

Models			Zero model (among all CTs)	Count model (among CTs with non-zero cycling infrastructure)
			N = 689	N = 576
Model 1-The quintile of material deprivation in 2011 as continues variable		Main effect	1.02 [0.68:1.52]	1.00 [0.95:1.05]
		Interaction effect	0.82 [0.30:2.22]	1.000 [0.99:1.00]
Model 2- The quintile of percentage of visible minorities in 2011 as continues variable		Main effect	0.62* [0.40:0.96]	0.99 [0.94:1.04]
		Interaction effect	0.71 [0.14:3.52]	1.00 [0.99:1.00]
Model 3- Gentrification status	Gentrified (2011–2016)	Main effect	1.20 [0.35:4.14]	1.11 [0.95:1.29]
		Interaction effect	0-	1.00 [0.99:1.01]
			–	
	Non-eligible for gentrification (2011–2016)	Main effect	0.60 [0.14:2.59]	0.77* [0.66:0.89]
		Interaction effect	0	1.01* [1.00:1.02]
			–	

Statistically significant values based on 95% confidence interval are denoted by an asterisk (*). CT refers to Census Tract. Zero and count models are based on hurdle modeling approach standing for the binary outcome (CTs with and without cycling infrastructure at baseline) and the continuous outcome (cycling infrastructure percentage among street length). Null values (zero) for interaction effects show that the model was not convergent.

## 4 Discussion

Inequities in access to cycling infrastructure have been well documented in the literature (Jahanshahi et al., 2021). A critique of this work is that most studies are cross-sectional, which limits our understanding of how changes in cycling infrastructure over time reduce or exacerbate inequities. Therefore, our study sought to examine how changes in cycling infrastructure between 2011 and 2016 were associated with CT-level material deprivation, visible minorities, and gentrification status in Montreal, Canada. In 2011, CTs with higher proportions of visible minorities or greater material deprivation had less cycling infrastructure. In CTs without any pre-existing cycling infrastructure in 2011, new infrastructure built between 2011 and 2016 was less abundant in CTs with more visible minorities. In CTs with pre-existing cycling infrastructure in 2011, non-eligible CTs for gentrification in 2011 (ie. those with higher median income) benefited from fewer cycling infrastructure expansions than non-gentrified CTs in Montreal between 2011 and 2016. Taken together, our study's findings contribute to the current understanding of inequities in cycling infrastructure and highlight the potential for longitudinal research to fully capture the dynamic relationships between cycling infrastructure, SES, and equity.

In 2011, disparities in cycling infrastructure were observed in certain Census Tracts (CTs), particularly those with higher proportions of visible minorities or greater material deprivation, which had less infrastructure compared to others. From 2011 to 2016, CTs with higher visible minority populations and no existing cycling infrastructure saw fewer new implementations. This suggests inequity in initial infrastructure installation. However, in CTs with pre-existing infrastructure, no significant overall change in equity structure was found. This underscores the need for continued efforts to promote equitable access to cycling infrastructure.

In line with our findings, a study conducted in 22 large US cities reported disparities in access to cycling infrastructure among CTs with different SES or demographic profiles in 2011. However, after five years, CTs with higher SES, greater educational attainment, and fewer Hispanic residents experienced more benefits from cycling infrastructure additions, resulting in increased, inequity (Braun et al., 2019). Similarly, a study conducted in 29 US cities found that although cycling infrastructure improvements primarily targeted lower-income CTs, those with a high proportion of people of color experienced the lowest rate of new cycling infrastructure installation. The study also revealed that socio-demographic changes led to the implementation of bicycle facilities, while implementation did not lead to socio-demographic change. In other words, bicycle facility investments did not contribute to residential mobility or relocation (Ferenchak & Marshall, 2021).

Our results highlight the importance of considering SES factors in the planning and implementation of cycling infrastructure projects to address inequities and ensure that all populations can benefit from the advantages of active transportation. This might include engaging and consulting with communities that are most impacted by transportation inequities, such as CTs with a higher percentage of people belonging to visible minorities and no pre-existing cycling infrastructure in Montreal, as our study showed that these CTs were actually less likely to receive new cycling infrastructure. Partnerships with community-based organizations, advocates, and other stakeholders can provide valuable insights and help to ensure that cycling infrastructure projects are implemented in a manner that is inclusive and equitable (Smith et al., 2009).

While the literature has shown that gentrification might be associated with increased cycling infrastructure in some context and has been known to exacerbate inequities (Cole et al., 2021; Stehlin, 2015), our study indicates that cycling infrastructure implementations in Montreal from 2011 to 2016 were not linked to gentrified status during that period. This is consistent with another study conducted in Montreal regarding simultaneous association between gentrification and cycling infrastructure (Kiani et al., 2023). However, that study has shown that gentrification between 2006 and 2011 contributed to an increase in cycling infrastructure between 2011 and 2016. It is worth mentioning that the mentioned study excluded ineligible CTs for gentrification and only compared gentrified and non-gentrified CTs. However, our study also considered ineligible for gentrification CTs (high-income CTs), which showed high-income CTs obtained smaller increases in cycling infrastructure compared to non-gentrified CTs between 2011 and 2016. As high-income CTs, had more cycling infrastructure in 2011, overall, cycling infrastructure interventions played a role in reducing inequities in cycling infrastructure between high-income CTs and non-gentrified CTs in Montreal between 2011 and 2016. It seems that gentrification is a process that unfolds over time, we recommend conducting longitudinal research to explore the relationship between gentrification and urban infrastructure change across different time periods.

In terms of methodology, we combined the use of a spatial mixed model framework with the hurdle approach to account for both the spatial autocorrelation in the data and the zero-inflated distribution of the outcome variable. We believe this method is a significant strength when modeling the association between urban change and SES factors. By accounting for the excess zeros in the data, we were able to produce more accurate estimates of the relationship between these two variables (Feng, 2021; Neelon et al., 2013). We recommend the hurdle approach as a useful methodology for future urban equity studies that investigate similar research questions, particularly when there are many CTs with null values or no change. Future studies can build on our findings by adopting the hurdle approach in their analysis to produce more accurate estimates of the association between cycling infrastructure change and SES factors in urban areas.

Our research has several limitations. We did not measure social conditions at the follow-up, which means we did not take into account any changes in SES factors between the baseline and follow-up. This limits our ability to more fully discuss the relationship between SES dynamics and cycling infrastructure change. Furthermore, this study is based solely on area-level data, and future research using individual-level data on cycling use and individual characteristics is necessary to provide a more nuanced understanding of how changes in cycling infrastructure may translate into actual bicycle usage and health benefits (Zhao & Manaugh, 2023). For example, multilevel modeling approaches could help identify the individual and contextual factors that influence cycling behavior. Use of mobility tracking and transportation mode detection could also be useful. Additionally, we acknowledge the potential value of future research endeavors that delve into the nuances of individual cycling infrastructure types. This avenue of inquiry holds promise for offering more refined insights into the varying levels of protection and planning requirements associated with distinct forms of cycling infrastructure. It is also noteworthy to mention that we incorporated both the quintiles of the percentage of visible minorities and material deprivation as continuous variables in our models. We explored the models using categorical variables, but encountered convergence issues, leading us to opt for the continuous approach. Our models also relied on one gentrification measure as proposed in the Genuine dataset using Ding's approach (Ding et al., 2016). Using other gentrification measures could generate different results (Firth et al., 2021). Finally, this is an association study, and we did not examine the contributing factors causing associations found in this study. For instance, it might be meaningful to consider the potential influence of road types on the associations we have observed between cycling infrastructure, SES factors, and gentrification. Particularly the prevalence of arterial roads in low SES areas, may act as a confounding or moderating factor. Arterial roads, designed to handle higher motor vehicle traffic volumes, may differ significantly from local roads in terms of their suitability for cycling infrastructure. Therefore, we excluded highways and maritime roads from the denominator in our models. Future research in this domain might consider contributing factors as potential covariates, allowing for a more nuanced understanding of how road infrastructure interacts with SES disparities and urban development dynamics to influence cycling infrastructure accessibility.

Based on our work, we would recommend the following practical steps in order to increase equity in cycling infrastructure in general and for Montreal:

1 Conduct a baseline equity analysis using key socio-demographic factors in the area of interest, and target areas and populations where infrastructure is lacking: In the case of Montreal, city policy could prioritize the implementation of cycling infrastructure in areas that have no existing infrastructure, especially those with higher proportions of visible minorities.
2 Monitor changes according to CTs' gentrification status: This study found that non-eligible CTs for gentrification benefited from fewer cycling infrastructure expansions than non-gentrified CTs in Montreal. City policy could monitor changes according to gentrification status and ensure that cycling infrastructure investments are not only distributed equitably, but benefit those that need it most.
3 Long-term planning for cycling infrastructure can help to ensure that equity is prioritized in infrastructure investments over time. This could include regular assessments of infrastructure gaps and needs and ensure that corrective measures are put in place to maintain equity as population distributions change over time.

## 5 Conclusion

Our study sheds light on local CT-level relationships between cycling infrastructure, SES factors, and gentrification. Our findings highlight the need to address historical inequities in the provision of cycling infrastructure in Montreal, particularly in CTs with higher levels of material deprivation and visible minorities. Despite these disparities, we found that increases in cycling infrastructure were not associated with SES factors. This means inequities may not be growing, but they do not seem to be corrected either. Overall, our study provides valuable insights for policymakers and researchers working to promote equitable access to active transportation options.

## Funding

This work was supported by the 10.13039/501100000024Canadian Institutes of Health Research under award number PJT-165955.

## Ethical Statement

Hereby, I, Behzad Kiani, consciously assure that for the manuscript Associations between gentrification, area-level socioeconomic status, and cycling infrastructure expansions in Montreal, Canada the following is fulfilled:

1) This material is the authors' own original work, which has not been previously published elsewhere.
2) The paper is not currently being considered for publication elsewhere.
3)The paper reflects the authors' own research and analysis in a truthful and complete manner.
4)The paper properly credits the meaningful contributions of co-authors and co-researchers.
5) The results are appropriately placed in the context of prior and existing research.
6) All sources used are properly disclosed (correct citation). Literally copying of text must be indicated as such by using quotation marks and giving proper reference.
7) All authors have been personally and actively involved in substantial work leading to the paper, and will take public responsibility for its content.

The violation of the Ethical Statement rules may result in severe consequences.

To verify originality, your article may be checked by the originality detection software iThenticate. See also http://www.elsevier.com/editors/plagdetect

I agree with the above statements and declare that this submission follows the policies of Solid State Ionics as outlined in the Guide for Authors and in the Ethical Statement.

Date: 28 April 2023.

Corresponding author's name: Behzad Kiani.

## CRediT authorship contribution statement

**Behzad Kiani:** Conceptualization, Formal analysis, Investigation, Methodology, Validation, Writing – original draft, Writing – review & editing. **Benoit Thierry:** Conceptualization, Formal analysis, Methodology, Software, Validation. **Philippe Apparicio:** Data curation, Resources. **Caislin Firth:** Conceptualization, Funding acquisition, Project administration. **Daniel Fuller:** Conceptualization, Funding acquisition, Supervision, Writing – review & editing. **Meghan Winters:** Conceptualization, Funding acquisition, Supervision, Writing – review & editing. **Yan Kestens:** Conceptualization, Data curation, Formal analysis, Funding acquisition, Investigation, Methodology, Resources, Supervision, Writing – review & editing.

## Declaration of competing interest

The authors declare no conflict of interest.

## Data Availability

Data will be made available on request.

## References

[bib1] Apparicio P., Carrier M., Gelb J., Séguin A.M., Kingham S. (2016). Cyclists' exposure to air pollution and road traffic noise in central city neighbourhoods of Montreal. Journal of Transport Geography.

[bib2] Bergmann vJ.von, Shkolnik D., Jacobs A. (2021). https://mountainmath.github.io/cancensus/index.html.

[bib3] Bivand R.R. (2022). Packages for analyzing spatial data: A comparative case study with areal data. Geographical Analysis.

[bib4] Braun L.M. (2021). Disparities in bicycle commuting: Could bike lane investment widen the gap?. Journal of Planning Education and Research.

[bib5] Braun L.M., Rodriguez D.A., Gordon-Larsen P. (2019). Social (in)equity in access to cycling infrastructure: Cross-sectional associations between bike lanes and area-level sociodemographic characteristics in 22 large U.S. cities. Journal of Transport Geography.

[bib6] Bruno M. (2022). Cycling and transitions theories: A conceptual framework to assess the relationship between cycling innovations and sustainability goals. Transportation Research Interdisciplinary Perspectives.

[bib7] Cole H.V.S., Mehdipanah R., Gullón P., Triguero-Mas M. (2021). Breaking down and building up: Gentrification, its drivers, and urban health inequality. Curr Environ Health Rep.

[bib8] Coxe S., West S.G., Aiken L.S. (2009). The analysis of count data: A gentle introduction to Poisson regression and its alternatives. Journal of Personality Assessment.

[bib9] de Oliveira C., Mason J., Jacobs R. (2021). Examining equity in the utilisation of psychiatric inpatient care among patients with severe mental illness (SMI) in Ontario, Canada. BMC Psychiatry.

[bib10] Ding L., Hwang J., Divringi E. (2016). Gentrification and residential mobility in philadelphia. Regional Science and Urban Economics.

[bib11] Doran A., El-Geneidy A., Manaugh K. (2021). The pursuit of cycling equity: A review of Canadian transport plans. Journal of Transport Geography.

[bib12] Equality in Access M. (2015). The case of bogotá’s sustainable transportation initiatives. Int J Sustain Transp.

[bib13] Evaluating active transport benefits and costs.

[bib14] Feng C.X. (2021). A comparison of zero-inflated and hurdle models for modeling zero-inflated count data. J Stat Distrib Appl.

[bib15] Ferenchak N.N., Marshall W.E. (2021). Bicycling facility inequalities and the causality dilemma with socioeconomic/sociodemographic change. Transportation Research Part Transp Environ.

[bib16] Firth C.L., Hosford K., Winters M. (2001). Who were these bike lanes built for? Social-Spatial inequities in vancouver’s bikeways. J Transp Geogr [Internet].

[bib17] Firth C., Thierry B., Fuller D., Winters M., Kestens Y. (2021). Gentrification, urban interventions and equity (GENUINE): A map-based gentrification tool for Canadian metropolitan areas. Health Reports.

[bib18] Fuller D., Gauvin L., Kestens Y., Morency P., Drouin L. (2013). The potential modal shift and health benefits of implementing a public bicycle share program in Montreal, Canada. International Journal of Behavioral Nutrition and Physical Activity.

[bib19] Government of Canada SC (2021). https://www23.statcan.gc.ca/imdb/p3Var.pl?Function=DEC&Id=45152.

[bib20] Hatzopoulou M., Weichenthal S., Dugum H., Pickett G., Miranda-Moreno L., Kulka R., Goldberg M. (2013). The impact of traffic volume, composition, and road geometry on personal air pollution exposures among cyclists in Montreal, Canada. Journal of Exposure Science and Environmental Epidemiology.

[bib21] Hoffmann M.L. (2013). https://www.proquest.com/docview/1429800527/abstract/7D20ED401F094E25PQ/1.

[bib22] Hosford K., Beairsto J., Winters M. (2022). Is the 15-minute city within reach? Evaluating walking and cycling accessibility to grocery stores in vancouver. Transportation Research Interdisciplinary Perspectives.

[bib23] Houde M., Apparicio P., Séguin A.M. (2018). A ride for whom: Has cycling network expansion reduced inequities in accessibility in Montreal, Canada?. Journal of Transport Geography.

[bib24] Jahanshahi D., Chowdhury S., Costello S.B., van Wee B. (2021). Review of key findings and future directions for assessing equitable cycling usage. Transportation Research Record.

[bib25] Jarry V., Apparicio P. (2021). Ride in peace: How cycling infrastructure types affect traffic conflict occurrence in montréal, Canada. Safety Now.

[bib26] Kiani B., Mamiya H., Thierry B., Firth C., Fuller D., Winters M., Kestens Y. (2023). The temporal sequence between gentrification and cycling infrastructure expansions in Montreal, Canada. Habitat International.

[bib27] Loignon C., Hudon C., Goulet É., Boyer S., De Laat M., Fournier N., Bush P. (2015). Perceived barriers to healthcare for persons living in poverty in quebec, Canada: The EQUIhealThY project. International Journal for Equity in Health.

[bib28] Lowan-Trudeau M., Keough N., Wong J., Haidey S. (2020). The affordable housing, transportation, and food nexus: Community gardens and healthy affordable living in Calgary. Can Geogr Géographies Can..

[bib29] Mölenberg F.J.M., Panter J., Burdorf A., van Lenthe F.J. (2019). A systematic review of the effect of infrastructural interventions to promote cycling: Strengthening causal inference from observational data. International Journal of Behavioral Nutrition and Physical Activity.

[bib30] Neelon B., Ghosh P., Loebs P.F. (2013). A spatial Poisson hurdle model for exploring geographic variation in emergency department visits. J R Stat Soc Ser A Stat Soc.

[bib31] Noyes P., Fung L., Lee K.K., Grimshaw V.E., Karpati A., DiGrande L. (2014). Cycling in the city: An in-depth examination of bicycle lane use in a low-income urban neighborhood. Journal of Physical Activity and Health.

[bib32] Padeiro M. (2022). Cycling infrastructures and equity: An examination of bike lanes and bike sharing system in Lisbon, Portugal. Cities Health.

[bib33] Padeiro M., Louro A., da Costa N.M. (2019). Transit-oriented development and gentrification: A systematic review. Transplantation Reviews.

[bib34] Pampalon R., Hamel D., Gamache P., Philibert M.D., Raymond G., Simpson A. (2012). An area-based material and social deprivation index for public health in Québec and Canada. Can J Public Health Rev Can Sante Publique.

[bib35] Parra D.C., Gomez L.F., Pinzon J.D., Brownson R.C., Millett C. (2018). Equity in cycle lane networks: Examination of the distribution of the cycle lane network by socioeconomic index in Bogotá, Colombia. Cities Health.

[bib36] Pebesma E. (2018). Simple features for R: Standardized support for spatial vector data. R J.

[bib37] Pratt R.H., Iv J.E.E., Levinson H.S., Turner S.M., Jeng C.Y., Nabors D. (2012). Chapter 16, pedestrian and bicycle facilities [internet].

[bib38] Pucher J., Buehler R. (2005). Cycling trends & policies in Canadian cities. World Transp Policy Pract [Internet].

[bib39] Pucher J., Buehler R. (2008). Making cycling irresistible: Lessons from The Netherlands, Denmark and Germany. Transplantation Reviews.

[bib40] V. Québec. Cycling in Québec in 2020 [Internet]. Vélo Québec; [cited 2023 Aug 21]. Available from:: https://www.velo.qc.ca/wp-content/uploads/2021/06/vq-edv2020-en.pdf.

[bib41] Rousset F., Ferdy J.B. (2014). Testing environmental and genetic effects in the presence of spatial autocorrelation. Ecography.

[bib42] Smith K.E., Bambra C., Joyce K.E., Perkins N., Hunter D.J., Blenkinsopp E.A. (2009). Partners in health? A systematic review of the impact of organizational partnerships on public health outcomes in England between 1997 and 2008. J Public Health Oxf Engl.

[bib43] Smith M., Hosking J., Woodward A., Witten K., MacMillan A., Field A., Mackie H. (2017). Systematic literature review of built environment effects on physical activity and active transport – an update and new findings on health equity. International Journal of Behavioral Nutrition and Physical Activity.

[bib44] Smithman M.A., Brousselle A., Touati N., Boivin A., Nour K., Dubois C.A., Breton M. (2018). Area deprivation and attachment to a general practitioner through centralized waiting lists: A cross-sectional study in quebec, Canada. International Journal for Equity in Health.

[bib45] Stehlin J. (2015). Cycles of investment: Bicycle infrastructure, gentrification, and the restructuring of the San Francisco bay area. Environ Plan Econ Space.

[bib46] Stein S. (2011). Bike lanes and gentrification: New York city’s shades of green. Progress Plan Mag [Internet], vol 188, 34-37.

[bib47] Strauss J., Miranda-Moreno L.F., Morency P. (2013). Cyclist activity and injury risk analysis at signalized intersections: A bayesian modelling approach. Accident Analysis & Prevention.

[bib48] Tortosa E.V., Lovelace R., Heinen E., Mann R.P. (2021). Infrastructure is not enough: Interactions between the environment, socioeconomic disadvantage, and cycling participation in England. J Transp Land Use.

[bib49] Tucker B., Manaugh K. (2018). Bicycle equity in Brazil: Access to safe cycling routes across neighborhoods in Rio de Janeiro and Curitiba. Int J Sustain Transp.

[bib50] Tulier M.E., Reid C., Mujahid M.S., Allen A.M. (2019). ‘Clear action requires clear thinking’: A systematic review of gentrification and health research in the United States. Health & Place.

[bib51] Vaswani M., Sutter A., Lapshina N., Esses V.M. (2023). Discrimination experienced by immigrants, racialized individuals, and indigenous peoples in small- and mid-sized communities in southwestern ontario. Can Rev Sociol Can Sociol.

[bib52] Vidal Tortosa E., Heinen E., Lovelace R., Heinen E., Götschi T. (2022). Advances in transport policy and planning [internet].

[bib53] Winters M., Fischer J., Nelson T., Fuller D., Whitehurst D.G.T. (2018). Equity in spatial access to bicycling infrastructure in mid-sized Canadian cities. Transportation Research Record.

[bib54] Zhao Q., Manaugh K. (2023). Introducing a framework for cycling investment prioritization. Transportation Research Record.

