# Supplementary material for: Associations between gentrification, census tract-level socioeconomic status, and cycling infrastructure expansions in Montreal, Canada
Source: SSM Popul Health. 2024 Feb 18;25:101637. doi: 10.1016/j.ssmph.2024.101637 (PMC10901850; doi:10.1016/j.ssmph.2024.101637)
Supplement: Multimedia component 1 [file mmc1.docx]

| Models with a social measure as the outcome | | OLS without spatial structures | OLS with spatial structure |
| --- | --- | --- | --- |
| Model 1- The quintile of material deprivation in 2011 as continues variable | | 0.89*  CI= [0.87 – 0.90] | 0.84*  CI= [0.76 – 0.93] |
| Model 2- The quintile of percentage of visible minorities in 2011 as continues variable | | 0.88*  CI= [0.86 – 0.89] | 0.87*  CI= [0.79 – 0.96] |
| Model 3- Gentrification status | Gentrified betw­een 2011 and 2016 | 1.13*  CI= [ 1.05: 1.21] | 1.00  CI= [0.74-1.35] |
|  | Ineligible for gentrification between 2011 and 2016 (High-income CTs) | 1.29*  CI= [1.23-1.36] | 1.16  CI = [0.85-.1.58] |

Table 1: Association between SES measures and cycling infrastructure in 2011

*OLS: Ordinary least square

Table 2: Association between SES measures in 2011 and cycling infrastructure in 2016, controlling for cycling infrastructure in 2011 to reflect the change in cycling infrastructure between 2011 and 2016

| Models | | | OLS without spatial structures | OLS with spatial structure |
| --- | --- | --- | --- | --- |
| Model 1-The quintile of material deprivation in 2011 as continues variable | | Main effect | 0.91*  [0.89:0.93] | 0.95  [0.89:1.00] |
|  |  | Interaction effect | 1.00*  [1.00:1.00] | 1.00  [0.99:1.00] |
| Model 2- The quintile of percentage of visible minorities in 2011 as continues variable | | Main effect | 0.95*  [0.93:0.98] | 0.92*  [0.86:.98] |
|  |  | Interaction effect | 1.00*  [1.00:1.00] | 1.00*  [1.00:1.00] |
| Model 3- Gentrification status | Gentrified (2011-2016) | Main effect | 1.23*  [1.12:1.35] | 1.00  [0.82:1.22] |
|  |  | Interaction effect | 0.99*  [0.99:0.99] | 1.00  [0.98:1.01] |
|  | Non-eligible for gentrification (2011-2016) | Main effect | 1.00  [0.87:1.00] | 0.79*  [0.65:0.97] |
|  |  | Interaction effect | 1.00  [0.99:1.00] | 1.01  [0.99:1.03] |

*OLS: Ordinary least square
